# Sustainable Valorization of Crickets: Optimized Low-Pressure Supercritical CO_2_ Extraction and the Oil’s Properties and Stability

**DOI:** 10.3390/foods15010114

**Published:** 2025-12-31

**Authors:** Dolaya Sadubsarn, Rattana Muangrat

**Affiliations:** 1 Division of Food Science and Technology, Faculty of Agro-Industry, Chiang Mai University, Chiang Mai 50100, Thailand; dolaya.sds@gmail.com; 2 Division of Food Process Engineering, Faculty of Agro-Industry, Chiang Mai University, Chiang Mai 50100, Thailand

**Keywords:** cricket oil, supercritical CO_2_, optimal extraction condition, antioxidant activity, oxidative stability

## Abstract

As part of the green valorization of crickets, cricket oil was extracted using supercritical CO_2_ at temperatures of 40–60 °C, pressures of 175–225 bar, and extraction times of 1–5 h to evaluate oil yield and physicochemical properties. Optimization was performed using Response Surface Methodology with a Box–Behnken Design. Oil yield ranged from of 9.35 to 16.19%, with acid values of 2.45–5.14 mg KOH/g oil, peroxide values of 20.06–70.34 mEq O_2_/kg oil, iodine values of 70.59–77.15 g I_2_/100 g oil, and saponification values of 178.07–196.76 mg KOH/g oil. Total phenolic content was 19.56–50.73 mg GAE/kg oil, and antioxidant activity measured by DPPH and ABTS assays ranged from 3.29 to 49.97 and from 36.82 to 145.90 mg Eq Trolox/kg oil, respectively. The main fatty acids were palmitic (27.36–28.84%), oleic (25.00–30.23%), linoleic (27.02–34.96%), and stearic acid (6.81–8.17%). The optimal extraction condition (60 °C, 200 bar, 5 h) yielded 15.86% SC-CO_2_-extracted cricket oil with favorable quality parameters, antioxidant activity, 1025 mg/100 g of cholesterol, and 14.9 mg/100 g of vitamin E. This oil was then used to study oxidative stability. With the addition of food-grade antioxidants (BHA, BHT, TBHQ, and DL-α-tocopherol at 75 mg/kg), TBHQ was the most effective in reducing oxidation, particularly at 45 and 55 °C. These findings demonstrate that supercritical CO_2_ extraction efficiently produces high-quality, solvent-free cricket oil with enhanced oxidative stability. Optimization of extraction temperature, pressure, and time identified suitable conditions that improved the oil’s physicochemical characteristics, supporting a sustainable and environmentally friendly extraction approach for cricket-based ingredients.

## 1. Introduction

The rising world population increases food demand; therefore, it is important to develop a new food supply that is safe, nutritious, and high-quality. Insects have gained attention due to their potential to be rich in nutrients and to produce lower levels of greenhouse gas emissions compared to traditional agriculture and livestock [1]. Insect production is increasing annually and is expected to reach 3,000,000 tons by 2030 [2]. Crickets are widely consumed across the world, especially in Asia and Africa. In Thailand, over 164 insect species are part of the diet, with *Acheta domesticus*, or the house cricket, being one of the most commonly consumed and cultivated [3]. Several studies, including those by Bawa et al. (2020) [4] and Pilco-Romero et al. (2023) [5], have reported the proximate composition of house crickets. For example, house crickets have been reported to contain approximately 48.06 to 76.19 g of protein, 8.9 to 43.9 g of fat, 3.70 to 7.50 g of fiber, 1.10 to 5.60 g of ash, and 1.60 to 10.20 g of carbohydrate per 100 g of dry sample. Variations in proximate composition have been attributed to differences in diet and the developmental stage of the insects [4,5]. Due to their rich nutritional profile, most research has focused on insect proteins, while their oils have received less attention. Insect oil is usually obtained as a by-product of protein extraction and is often discarded. Therefore, to increase its value, research should focus on oil quality and its potential as an alternative edible oil in food applications.

Regarding house cricket oil, the main fatty acids are saturated fatty acids (such as stearic and palmitic acids) and unsaturated fatty acids (such as linoleic and oleic acids) [5], with linoleic acid being an essential fatty acid that cannot be synthesized by the human body [6]. Due to its fatty acid composition, house cricket oil may have the potential to serve as a health-promoting fat source, as linoleic and oleic acids can reduce the risk of cardiovascular disease by lowering low-density lipoprotein (LDL) cholesterol and increasing high-density lipoproteins (HDLs) [5]. Moreover, crickets have been reported to contain a higher level of linoleic acid than other insects, such as locusts and silkworms [7]. Therefore, further research and development of house cricket oil are needed to add value to cricket oil.

Oil extraction methods play a crucial role in determining both oil yield and quality. Previous studies have reported the extraction of oil from edible insects, including crickets, using mechanical pressing and organic solvent extraction techniques [8,9,10,11,12,13,14]. Mechanical extraction, particularly screw pressing, has been applied due to its simplicity, low cost, and solvent-free operation, and has been used for cricket oil recovery [9,11]. However, this method generally results in moderate oil yields and may produce fine solid residues in the extracted oil, which can affect oil clarity and quality [9]. Organic solvent extraction, such as hexane [11] or chloroform–methanol mixtures [13,14], can also be applied to extract cricket oil. Furthermore, techniques such as ultrasonication [11] and high hydrostatic pressure (HHP) [12]-assisted solvent extraction have been used to improve oil recovery. Nevertheless, concerns remain regarding residual solvents, environmental impact, safety, and disposal [8]. Both mechanical pressing and solvent extraction, with or without assisted technologies, influence oil yield and fatty acid composition in crickets [9,11,14].

In recent years, supercritical carbon dioxide (SC-CO_2_) extraction has emerged as an environmentally friendly and solvent-free alternative for lipid recovery. This technique operates under relatively mild temperatures, leaves no solvent residues, and enables efficient oil extraction while preserving bioactive compounds. SC-CO_2_ extraction has been successfully applied to obtain oil from various edible insects, including *Gryllus bimaculatus*, *Tenebrio molitor* larvae, *Hermetia illucens* larvae, *Locusta migratoria*, and *Zophobas atratus* larvae [10,15,16,17,18]. A comparative study reported no significant differences in crude oil yield or fatty acid composition between SC-CO_2_ extraction and conventional hexane extraction for house crickets and mealworms, demonstrating the feasibility of this technique for insect oil recovery [10]. However, information on the optimization of SC-CO_2_ extraction conditions for house cricket oil remains limited, particularly regarding the combined effects of temperature, pressure, and extraction time on oil yield and physicochemical properties. Further optimization of SC-CO_2_ extraction conditions represents a promising research direction, as information on how extraction parameters influence the oil yield and quality of cricket oil remains limited, especially under mild, sustainable, and environmentally friendly processing conditions.

Even though cricket oil contains fatty acids that are beneficial to human health, its high degree of unsaturation makes it more prone to oxidation. Lipid oxidation produces peroxides and hydroperoxides that shorten shelf life and reduce oil quality. To prevent this, food-grade antioxidants, such as butylated hydroxytoluene (BHT), butylated hydroxyanisole (BHA), tert-butylhydroquinone (TBHQ), and α-tocopherol, can prevent the chain reactions of lipid peroxidation by scavenging free radicals, thereby preserving the oil’s quality and extending its shelf life [19]. Therefore, this study aimed to (1) determine suitable extraction conditions for *A. domesticus* using SC-CO_2_ at low extraction pressure to obtain a high crude oil yield, with the effects of extraction pressure, extraction temperature, and extraction time evaluated through Response Surface Methodology (RSM); (2) analyze the physicochemical properties of SC-CO_2_-extracted cricket oil, including acid value (AV), peroxide value (PV), iodine value (IV), saponification value (SV), total phenolic content (TPC), antioxidant activities (ABTS and DPPH radical scavenging), vitamin E, and cholesterol; and (3) assess the oxidative stability of the extracted cricket oil during storage by adding food-grade antioxidants including BHA, BHT, TBHQ, and DL-α-tocopherol.

## 2. Materials and Methods

### 2.1. Sample Preparation

House crickets were purchased in boiled and frozen form from a local farmer (Organic Agriculture Community Enterprise Group in Ban Mae Tat, Huai Sai, Chiang Mai, Thailand). Prior to extraction, the crickets were thawed and dried in a tray dryer at 65 °C for 17 h until the moisture content was reduced to below 5%. The dried samples were then ground to reduce particle size, resulting in a median particle size (D_50_) of approximately 1.0 mm, as determined from the cumulative particle size distribution. The ground samples were sealed in bags and stored at room temperature until oil extraction. The physicochemical properties of cricket oil obtained by supercritical CO_2_ extraction were subsequently compared with those of commercially available screw-pressed cricket oil (JR Unique Foods Ltd., Udon Thani, Thailand).

### 2.2. Optimization of Cricket Oil Extraction Using SC-CO_2_

#### 2.2.1. Cricket Oil Extraction Using the Soxhlet Extraction Method

Cricket oil was extracted using the Soxhlet extraction method. A 5 g sample was placed in a thimble and extracted with hexane for 6 h. During the process, hexane vapor repeatedly condensed and washed the sample, dissolving the oil, which was collected in the flask. After extraction, the solvent was evaporated to obtain the extracted oil. The oil yield was measured, and its physicochemical properties were subsequently analyzed.

#### 2.2.2. Cricket Oil Extraction Using SC-CO_2_

The extraction system used was a supercritical CO_2_ extractor specified by the manufacturer as a 5 L unit (Guangzhou Heavensent Industrial Co., Ltd., Guangzhou, China). The extraction vessel column employed in this study had an effective internal volume of 3.86 L, with an internal diameter of approximately 9.50 cm and an external diameter of 10.50 cm. The internal and external heights of the column were 54.50 and 58.50 cm, respectively. The dried cricket samples (500 g) were first ground to reduce particle size, resulting in a median particle size (D_50_) of approximately 1.0 mm, as determined from the cumulative particle size distribution. The ground samples were then loaded into the extraction column to a bed height of approximately 15.0 cm. This bed height was selected to allow uniform penetration of supercritical CO_2_ through the samples and to avoid excessive pressure drop. The porosity, real density, and apparent density of the packed bed were approximately 0.596, 1.174 g/cm^3^, and 0.474 g/cm^3^, respectively. Carbon dioxide with a purity of 99.9% was used, and the flow rate was continuously adjusted during extraction to maintain constant pressure. Extraction times of 1, 3, and 5 h were selected based on preliminary experiments, as the oil yield reached a plateau within this time range at pressures of 175 to 225 bar, and extending the extraction time beyond 5 h did not result in further increases in oil yield.

After extraction, the oil was separated in the collection vessel, collected in amber glass bottles, flushed with nitrogen to minimize oxidation, and stored at 4 °C until further analysis. Each extraction condition was performed in triplicate. Crude oil yield (%) was calculated according to Equation (1).

(1) $$\mathrm{Crude}\;\mathrm{oil}\;\mathrm{yield}(\%)=\frac{w_{o}}{w_{s}}\times 100$$

where $w_{o}$ is the weight of extracted cricket oil (g), and $w_{s}$ is the weight of ground cricket sample used for extraction (g).

#### 2.2.3. Experimental Design

A Box–Behnken design (BBD) was applied using Minitab version 16 to generate the experimental treatments, and Response Surface Methodology (RSM) was employed to evaluate the influence of extraction parameters on the process. A total of fifteen experimental treatments were generated based on three levels for each parameter (Table 1). The temperature (X_1_) was set at 40, 50, and 60 °C; pressure (X_2_) at 175, 200, and 225 bar; and time (X_3_) at 1, 3, and 5 h. The experimental data were fitted to a quadratic polynomial model as described in Equation (2).

(2) $$Y=b_{0}+\sum_{i=1}^{k}b_{i}X_{i}+\sum_{i=1}^{k}b_{ii}X_{i}^{2}+\sum_{i\neq j=1}^{k}b_{ij}X_{i}X_{j}$$

where Y represents the response, b_0_ is a constant term, b_i_ denotes the linear regression coefficient for main variable effects, $b_{ii}$ represents the quadratic coefficient, and b_ij_ is the coefficient for interaction effects, while X_i_ and X_j_ are the uncoded independent variables. Model validation was performed by repeating the experiments three times and comparing the observed values with the predicted results to assess accuracy.

The experimental data generated from the Box–Behnken design were used to develop a quadratic polynomial regression model describing the relationship between extraction variables and oil yield. This model was subsequently applied to determine the optimal extraction conditions. Model validation was performed by conducting additional extraction experiments in triplicate under conditions close to the predicted optimal extraction condition, and the experimental results were compared with the model predictions to evaluate accuracy.

### 2.3. Physicochemical Analysis of Extracted Cricket Oil Using SC-CO_2_

#### 2.3.1. Determination of Acid Value and Percentage of Free Fatty Acids

Acid value (AV) was determined according to AOAC Official Method 940.28 [20]. Briefly, 2 g of oil sample was weighed into a 250 mL Erlenmeyer flask, followed by the addition of 50 mL ethanol and 1 mL phenolphthalein indicator. The mixture was boiled for 5 min and titrated while still hot with 0.1 N NaOH until a persistent pink endpoint was observed. The volume of NaOH was recorded, and AV was calculated using Equation (3).

(3) $$AV\;(mg\;KOH/g\;oil)=\frac{56.1\times V\times N}{W}$$

where V is the volume of titrated NaOH (mL), N is the concentration of titrated NaOH (N), and W is the sample weight (g).

#### 2.3.2. Determination of Peroxide Value

Peroxide value (PV) was determined following AOAC Official Method 965.33 [20]. A 2 g oil sample was weighed into a 250 mL Erlenmeyer flask and mixed with 30 mL of 3:2 (*v*/*v*) acetic acid/chloroform. Then, 500 µL of saturated KI were added, shaken for 1 min, and incubated in the dark for 5 min. Afterward, 30 mL of distilled water was added into the mixture, which was then titrated with 0.01 N Na_2_S_2_O_3_, and 500 µL of starch solution was added as an indicator. The titration continued with 0.01 N Na_2_S_2_O_3_ until the blue color disappeared. The volume of Na_2_S_2_O_3_ used was recorded. PV was calculated using Equation (4).

(4) $$PV\;(mEq\;O_{2}/kg\;oil)=\frac{(V-V_{b})\times N\times 1000}{W}$$

where V is the volume of titrated Na_2_S_2_O_3_ (mL), $V_{b}$ is the volume of Na_2_S_2_O_3_ for the titration blank (mL), N is the concentration of Na_2_S_2_O_3_ (N), and W is the sample weight (g).

#### 2.3.3. Determination of Iodine Value

Iodine value (IV) was determined based on AOAC Method 920.158 [20]. A 0.5 g oil sample was added to a 250 mL Erlenmeyer flask and mixed with 15 mL of a 1:1 (*v*/*v*) cyclohexane/acetic acid solution. Then, 25 mL of WIJS solution was added, and the mixture was incubated in the dark for 1 h. After incubation, 20 mL of 15% KI solution and 150 mL of distilled water were added, followed by titration with 0.1 N Na_2_S_2_O_3_ and the addition of 500 µL of starch indicator. The titration with 0.1 N Na_2_S_2_O_3_ continued until the blue color disappeared. The volume of Na_2_S_2_O_3_was recorded. IV was calculated using Equation (5).

(5) $$IV\;(g\;I_{2}/100\;g\;oil)=\frac{(V_{b}-V)\times N\times 12.6}{W}$$

where V is the volume of titrated Na_2_S_2_O_3_ (mL), $V_{b}$ is the volume of Na_2_S_2_O_3_ for the titration blank (mL), N is the concentration of Na_2_S_2_O_3_ (N), and W is the sample weight (g).

#### 2.3.4. Determination of Saponification Value

Saponification value (SV) was measured following AOAC Standard Method 920.160 [20]. An oil sample (1.5–2 g) was added to a 250 mL round-bottom flask and mixed with 25 mL of 4% ethanolic KOH. The mixture was refluxed for 30 min and cooled to room temperature. After cooling, 1 mL of phenolphthalein indicator was added, and the mixture was titrated with 0.5 N HCl until the pink color disappeared. The volume of HCl was recorded. The blank was determined under the same procedure without adding the oil sample. The SV was calculated using Equation (6).

(6) $$SV\;(mg\;KOH/g\;oil)=\frac{56.1(V_{b}-V)\times N}{W}$$

where V is the volume of titrated HCl (mL), $V_{b}$ is the volume of HCl for the titration blank (mL), N is the concentration of HCl (N), and W is the sample weight (g).

#### 2.3.5. Measurement of Cricket Oil Viscosity

Cricket oil viscosity was determined using a Brookfield RVDV-II+ viscometer (Brookfield, Middleboro, MA, USA). The sample (8 mL) was tempered at 25 °C and transferred to the sample vessel. The viscosity was measured with spindle no. 18 at a rotational speed of 180 rpm. The viscosity values were recorded after 2 min in centipoise (cP).

#### 2.3.6. Measurement of Cricket Oil Color

Oil color was determined with a ColorQuest XE spectrophotometer (HunterLab, Reston, VA, USA). For each measurement, 20 mL of oil sample was filled in a cuvette and analyzed using the TTRAN mode. Color parameters were reported in the CIE Lab coordinates, where L* represents lightness, a* denotes the red–green axis, and b* is the yellow–blue axis.

#### 2.3.7. Determination of Oil Specific Gravity

Specific gravity was determined according to AOAC Method 920.212 [20] using a pycnometer. A clean and dry pycnometer was weighed and recorded as $W_{1}$. It was then filled with distilled water and tempered in a water bath at 25 °C for 30 min, and the weight was recorded again as $W_{2}$. After cleaning and drying, it was filled with an oil sample, placed in a water bath at 25 °C for 30 min, and weighed to obtain $W_{3}$. The specific gravity of the oil was calculated using Equation (7).

(7) $$Specific\;gravity=\frac{W_{3}-W_{1}}{W_{2}-W_{1}}$$

#### 2.3.8. Smoke Point

The smoke point of the oil was measured according to AOCS Official Method Cc 9a-48 [21]. Oil was poured into the test cup to the marked filling line. The test cup was placed so that the light beam was centered, and the thermometer was set at the center of the cup with its bulb positioned 6.5 mm above the cup bottom. The sample was first heated rapidly until it was within 42 °C of the expected smoke point, after which the heating rate was adjusted to 5–6 °C per min. The smoke point was recorded when the first bluish smoke stream appeared.

### 2.4. Determination of Total Phenolic Content

Total phenolic content (TPC) was analyzed using the Folin–Ciocalteu method modified from a previous report [22]. Cricket oil (1 g) was mixed with 1 mL of hexane and 2 mL of 80% (*v*/*v*) methanol, vortexed for 15 s, and centrifuged at 4500 rpm for 10 min. Then, 1 mL of the methanol phase was taken and mixed with 5 mL of 10% (*v*/*v*) Folin–Ciocalteu reagent, vortexed, and incubated in the dark for 5 min. Subsequently, 1 mL of 20% (*w*/*v*) Na_2_CO_3_ was added to the mixture. The mixture solution was incubated at room temperature in the dark for 1 h. Absorbance was measured at 725 nm against a blank (1 mL of distilled water, 5 mL of 10% (*v*/*v*) Folin–Ciocalteu solution, and 1 mL of 20% (*w*/*v*) Na_2_CO_3_). TPC was quantified using a gallic acid standard curve and expressed as mg GAE/kg oil.

#### Analysis of Individual Phenolic Compounds by HPLC

A 20 µL aliquot of extracted oil was mixed with 800 µL of isopropanol and vortexed until homogeneous. The mixture was then diluted with 99.99% acetonitrile at a ratio of 1:1 (*v*/*v*), centrifuged at 13,000 rpm for 5 min, and the supernatant was filtered through a 0.45 µm PTFE syringe filter. The filtrate was transferred into HPLC vials for subsequent analysis. Chromatographic separation was performed using a C18-ODS column (250 mm × 4.6 mm, 5 µm). The mobile phase consisted of solvent A (2% acetic acid in water), solvent B (100% acetonitrile), solvent C (deionized water), and solvent D (100% methanol). The flow rate was set at 1.0 mL/min, the column temperature was maintained at 30 °C, and the injection volume was 10 µL. Detection was carried out using a diode-array detector (DAD) at 280 nm. The total run time was 85 min, and the needle wash solution was 10% acetonitrile.

Individual phenolic compounds were identified by comparing their retention times and UV absorbance profiles obtained from the DAD at 280 nm with those of authentic external standards analyzed under identical chromatographic conditions. The phenolic standards used in this study included gallic acid, theobromine, protocatechuic acid, p-hydroxybenzoic acid, catechin, chlorogenic acid, caffeine, vanillic acid, caffeic acid, syringic acid, epicatechin, vanillin, p-coumaric acid, ferulic acid, sinapic acid, rutin, myricetin, trans-cinnamic acid, and quercetin. Standard solutions of these phenolic compounds were prepared at concentrations not exceeding 100 ppm.

Quantification of individual phenolic compounds was performed by comparing the peak areas of the identified compounds in the samples with those of the corresponding external standards.

### 2.5. Determination of DPPH Radical Scavenging Activity

DPPH radical scavenging activity was determined using a modified method adapted from [23]. A 0.3 g of cricket oil was mixed with 4.5 mL of methanol and vortexed for 15 s, followed by centrifugation at 4500 rpm for 10 min. Subsequently, 1 mL of the methanolic phase was mixed with 100 µL of 1 mM DPPH solution (prepared in methanol). The mixture was vortexed, incubated in the dark for 10 min, and the absorbance was measured at 515 nm. Antioxidant activity was calculated against a Trolox standard curve and reported as mg Trolox equivalents per kg of oil.

### 2.6. Determination of ABTS Radical Scavenging Activity

ABTS radical scavenging activity was determined according to a previously described method with modifications from [24]. The ABTS solution was prepared by reacting 7 mM ABTS with 2.45 mM potassium persulfate in the dark at room temperature for 16 h and then diluted with methanol to an absorbance of 0.70 ± 0.02 at 734 nm. For analysis, 0.3 g of cricket oil was mixed with 4.5 mL of methanol, vortexed for 15 s, and centrifuged at 4500 rpm for 10 min. A 100 µL of the methanolic phase was added to 1000 µL of the prepared ABTS solution, vortexed, and incubated in the dark for 5 min. The absorbance was then measured at 734 nm. Antioxidant activity was determined using a Trolox calibration curve and expressed as mg Trolox equivalents per kg of oil.

### 2.7. Determination of Fatty Acids Composition

Fatty acid composition was determined according to the methods of Folch et al. (1957) and Morrison and Smith (1964) [25,26]. Briefly, 0.5 g of oil was weighed into a round-bottom flask, mixed with 5 mL of 0.5 M NaOH, and refluxed at 110–120 °C for 5 min for saponification. Subsequently, 5 mL of 20% boron trifluoride in methanol was added, and the mixture was refluxed for an additional 5 min to convert fatty acids into their corresponding fatty acid methyl esters (FAMEs). After cooling, the reaction mixture was transferred to a separatory funnel, followed by the addition of 5 mL hexane and 10 mL of saturated NaCl solution. The mixture was shaken and allowed to separate, and the upper hexane layer containing FAMEs was collected and filtered through a 0.45 µm nylon membrane filter into GC vials. FAMEs were analyzed using a gas chromatograph (Shimadzu Nexis GC-2030, Kyoto, Japan) equipped with a flame ionization detector (FID) and an RT^®^-2560 capillary column (100 m × 0.25 mm ID, 0.2 µm film thickness; Restek^®^, Bellefonte, PA, USA). The injector and detector temperatures were set at 225 °C and 250 °C, respectively. The column oven temperature was programmed from 100 °C (held for 4 min) to 240 °C at a rate of 3.5 °C/min. Helium was used as the carrier gas at a constant flow rate of 1 mL/min.

Individual fatty acids were identified by comparing their retention times with those of a fatty acid methyl ester (FAME) external standard mixture (Supelco 37 component FAME Mix, Merck, Darmstadt, Germany) analyzed under identical chromatographic conditions. Quantification was performed by comparing the peak areas of individual fatty acids in the samples with those of the corresponding external standards.

### 2.8. Determination of Vitamin E

Vitamin E content in SC-CO_2_-extracted cricket oil was analyzed by an external laboratory. A 300 g sample of extracted cricket oil was delivered to ALS Laboratory Group (Thailand) Co., Ltd. (Bangkok, Thailand). Vitamin E was determined using the in-house method STM No.03-021 [27], which provides the following analytical details:

A 3 g sample of cricket oil was weighed into a 125 mL amber Erlenmeyer flask, and 50 mg of pyrogallic acid was added as an antioxidant. To this flask, 22 mL of 95% ethanol and 8 mL of ethanolic KOH (0.25 g/mL) were added. The mixture was refluxed under a nitrogen atmosphere with occasional swirling for 45 min. After cooling to room temperature, the reaction mixture was neutralized with 20 mL of acetic acid in 95% ethanol and quantitatively transferred to a 100 mL amber volumetric flask. The volume was adjusted to 100 mL with ethanol and mixed thoroughly. The solution was filtered through a 0.45 μm filter, and a 20 μL aliquot was injected into the HPLC system. Separation was performed using methanol–water (95:5, *v*/*v*), adjusted to pH 4.0 with acetic acid, at a flow rate of 1.5 mL/min. α-Tocopherol eluted at approximately 10 min. Calibration was carried out with vitamin E standards, and concentrations in samples were determined by comparing peak heights or areas with those of the standards.

### 2.9. Determination of Cholesterol

Cholesterol content in SC-CO_2_-extracted cricket oil was analyzed by an external laboratory. A 300 g sample of cricket oil was shipped to ALS Laboratory Group (Thailand) Co., Ltd. (Bangkok, Thailand). The analysis was conducted according to the laboratory’s in-house method STM No. 03-027, which was adapted from the procedure of Al-Hasani et al. [28], which provides the following analytical details:

A 5–10 g sample was saponified with 1 mL of 60% KOH/gram of sample under reflux for 30 min and then cooled to room temperature. The mixture was combined with 100 mL of hexane, stirred for 10 min, followed by the addition of 25 mL of deionized water and stirring for another 15 min. After phase separation, the hexane layer was collected, passed through anhydrous sodium sulfate to remove residual moisture, and transferred into a clean flask. A 25 mL aliquot of the hexane extract was evaporated to dryness using a gentle flow of nitrogen, and the residue was dissolved in 2 mL of hexane containing 0.2 mg/mL 5α-cholestane as the internal standard. The prepared solution was transferred to a vial, and 5 µL was injected into the gas chromatograph. Cholesterol was quantified based on a retention time of approximately 7 min relative to the internal standard at approximately 4 min, and results were expressed as mg/100 g oil.

### 2.10. Determination of Oxidative Stability of Cricket Oil

The cricket oil extracted under the conditions that provided the highest oil yield, as obtained in Section 2.2, was selected for the oxidative stability study. Antioxidants, including BHA, BHT, TBHQ, and DL-α-tocopherol, were added to the extracted cricket oil at an equal final concentration of 75 mg/kg. The oil samples were stored in brown glass bottles, flushed with N_2_, and kept at 25 °C, 45 °C, and 55 °C for 60 days. In this study, oxidative stability during storage was evaluated using the PV, with samples collected and measured on days 0, 5, 10, 15, 30, and 60.

### 2.11. Statistical Analysis

Each extraction run was performed in triplicate, and all quality analyses of the extracted oil were also measured in triplicate. Data were analyzed using analysis of variance (ANOVA), and mean differences were evaluated with Duncan’s multiple range test (DMRT) at a significance level of *p* < 0.05, using SPSS software (Version 17.0). Three-dimensional (3D) response surface plots were generated from the quadratic polynomial regression model (Equation (2)) using Minitab version 16 and SigmaPlot version 15.0.

## 3. Results

### 3.1. Physical and Chemical Properties of Soxhlet-Extracted Cricket Oil

The results of the physical and chemical analysis of cricket oil obtained using Soxhlet extraction are shown in Table 2. Cricket oil extracted by the Soxhlet method showed an oil yield of 15.75%. The acid value (11.54 mg KOH/g oil) and peroxide value (40.04 mEq O_2_/kg oil) indicated that the extracted oil was susceptible to hydrolytic and oxidative changes during processing. The iodine value (68.75 g I_2_/100 g oil) reflected a moderate degree of unsaturation, while the saponification value (168.41 mg KOH/g oil) suggested the presence of medium-chain fatty acids. In terms of physical properties, the oil exhibited a viscosity of 64.18 cP and a density of 0.901 g/cm^3^, which are consistent with edible insect oils reported in previous studies. It should be noted that Soxhlet extraction requires an additional solvent evaporation step to obtain the final oil, which can be time-consuming and may affect oil quality. To overcome this limitation, the present research also employed supercritical CO_2_ extraction, a solvent-free method that eliminates the evaporation step. The findings of this approach are presented in the following section.

### 3.2. Optimization of Cricket Oil Extraction Using Supercritical CO_2_

The cricket sample used in this study contained 3.56 ± 0.10% moisture, 16.20 ± 0.34% crude fat, 11.23 ± 0.47% crude fiber, 61.08 ± 0.79% protein, 4.19 ± 0.09% ash, and 3.72 ± 0.93% carbohydrate. Crude oil yield obtained from SC-CO_2_ extraction ranged from 9.35 to 16.19%, with the maximum yield of crude oil (16.19%) obtained under the extraction conditions of 60 °C, 200 bar, and 5 h (Table 3). Moreover, crude oil yields obtained under the extraction conditions of 60 °C and 225 bar for 3 h, 50 °C and 175 bar for 5 h, and 50 °C and 200 bar for 3 h did not differ significantly (*p* > 0.05). In contrast, the lowest oil yield (9.35%) was obtained under the condition of 50 °C, 175 bar, and 1 h, which could be due to the lower extraction pressure and shorter extraction time. Mass balance values for SC-CO_2_ extraction were consistently close to 100% (97.84–102.05%), confirming that the extraction process has high efficiency.

The experimental data were analyzed using a quadratic polynomial model, and the significance of coefficients was tested by ANOVA. Non-significant terms were excluded from the analysis, as shown in Table 4. The predictive model equation in uncoded variables was generated using Minitab version 16 and is shown in Equation (8):

(8) Y = −68.7657 − 0.3115X_1_ + 0.7344X_2_ + 8.9793X_3_ + 0.0034X_1_^2^ − 0.0015X_2_^2^ − 0.2269X_3_^2^ − 0.0351X_2_X_3_

where Y represents the predicted crude cricket oil (%), X_1,_ X_2,_ and X_3_ represent the extraction parameters: temperature (°C), pressure (bar), and time (h), respectively.

From Equation (8), pressure and time had significant positive effects on crude oil yield, while temperature showed a negative effect (*p* < 0.05). The quadratic terms (X_2_^2^ and X_3_^2^) indicated that increasing pressure and extraction time could reduce oil yield. However, the positive coefficient of temperature (X_1_^2^) suggests that increasing temperature can enhance the yield. The interaction between pressure and extraction time (X_2_X_3_) had a significant negative effect on oil yield (*p* < 0.05). The predicted equation had R^2^_adj_ of 93.78% with a lack-of-fit *p*-value of 0.332 (as shown in Table 4), confirming that the quadratic model adequately described the experimental data and reliably predicted the response.

### 3.3. Response Surface Analysis of Cricket Oil Yield Obtained from Supercritical CO_2_ Extraction

**Figure 1:**
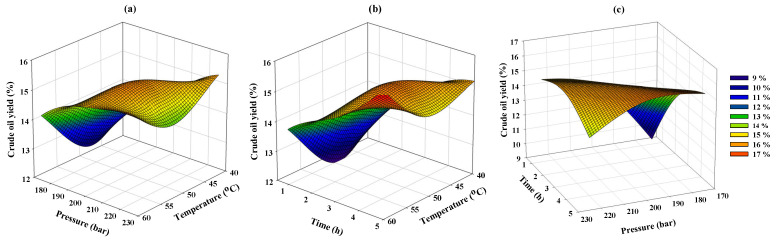
Response surface plots showing the effects of extraction temperature, extraction pressure, and extraction time on crude oil yield during supercritical CO_2_ extraction: (**a**) extraction pressure and temperature; (**b**) extraction temperature and time; and (**c**) extraction pressure and time.

RSM was applied to analyze the effects of extraction parameters on crude oil yield, for which surface plots were generated by evaluating the interaction of two parameters while keeping one parameter constant. Figure 1a illustrates the effect of pressure and temperature on crude cricket oil yield. Crude oil yield increased at high pressure and high temperature. This may be attributed to higher pressure increasing solvent density and improving its ability to dissolve oil from the solid matrix, while higher temperature increases the vapor pressure of the solutes and improves solvent diffusivity, leading to enhanced extraction of oil into the SC-CO_2_ phase [17,29,30,31]. Figure 1b demonstrates the effect of extraction time and extraction temperature on crude cricket oil yield. As both extraction time and extraction temperature increase, crude oil yield also increases. However, extraction time showed a stronger influence and more significantly increased crude oil yield, as reported in Equation (8) (*p* < 0.05). Figure 1c shows that extraction pressure and extraction time positively affect crude oil yield. This trend aligned with previous studies [16,17], which reported that higher pressure improves insect oil yield. This could be due to higher pressure increasing the solubility of oil in SC-CO_2_ [29]. However, it was observed that longer extraction times and higher pressures did not further improve crude oil yield, indicating that the extractable oil had already been recovered under those conditions.

### 3.4. Physicochemical Analysis of Extracted Cricket Oil Using Supercritical CO_2_

**Figure 2:**
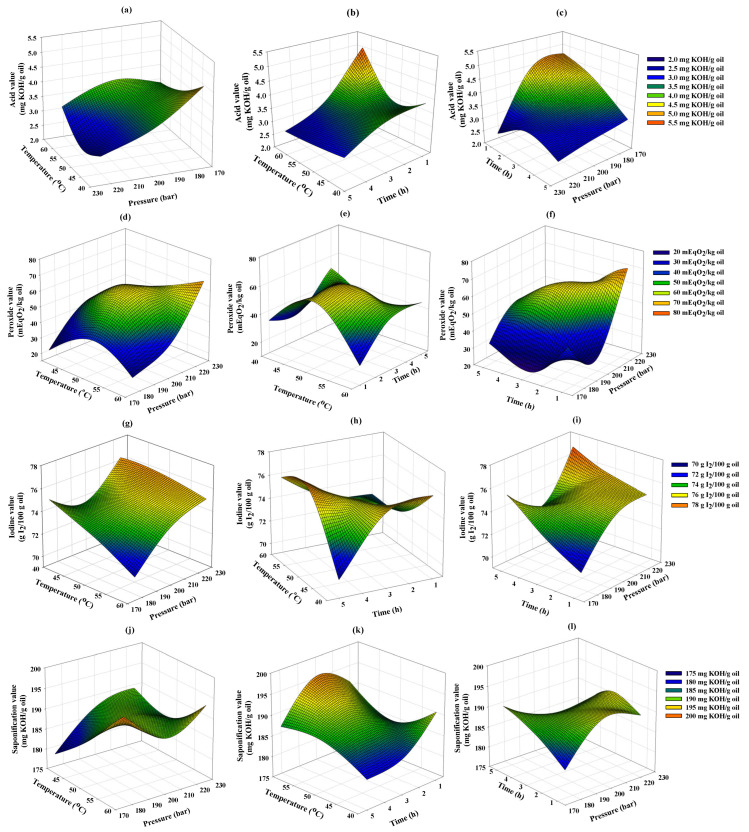
Response surface plots showing the effects of extraction parameters including, temperature, pressure, and time on (**a**–**c**) acid value (AV), (**d**–**f**) peroxide value (PV), (**g**–**i**) iodine value (IV), and (**j**–**l**) saponification value (SV) of the supercritical CO_2_-extracted cricket oil.

The physicochemical properties of SC-CO_2_-extracted cricket oil (AV, PV, IV, and SV) were analyzed and compared with screw-pressed commercial cricket oil (JR Unique Foods Ltd., Udon Thani, Thailand) (as shown in Table 5 and Table 6). RSM was applied to investigate the effects of extraction temperature, pressure, and time on the oil quality, and the 3D surface plots are displayed in Figure 2.

#### 3.4.1. Acid Value and Percentage of Free Fatty Acids

AV measures the free fatty acids (FFAs) in oil, with higher values indicating oil deterioration [32]. The AVs of SC-CO_2_-extracted cricket oils varied from 2.45 to 5.14 mg KOH/g oil. Figure 2a–c demonstrate the effect of extraction parameters (temperature, pressure, and time) on AV. An increase in temperature at constant pressure led to a higher AV. This could be due to high temperature increasing the volatilization and solubility of free fatty acids, resulting in an increase in AV [33]. In contrast, AV seemed to decrease at higher pressure, as higher pressure improves the CO_2_ density and its solvent power and selectivity toward non-polar triglycerides, resulting in lower AV [33]. This behavior is consistent with previous studies, reporting that higher pressure led to a reduction in AV in canola oil and lipid extraction from coffee silverskin using SC-CO_2_ [33,34]. In addition, AV tended to decrease with longer extraction time, possibly because increased extraction time allows more triglycerides to be extracted, thereby diluting the relative concentration of FFAs.

The SC-CO_2_-extracted cricket oil exhibited lower AV than screw-pressed commercial cricket oil (5.17 mg KOH/g oil). The lower AV of SC-CO_2_-extracted oil could be due to the non-polar properties of SC-CO_2_, which allow effective extraction of lipids while minimizing the extraction of FFAs [35]. Additionally, the majority of AVs obtained for cricket oil extracted by SC-CO_2_ exhibited AVs below 4 mg KOH/g oil, indicating good stability in accordance with the Codex Alimentarius standard [36].

#### 3.4.2. Peroxide Value

**Figure 3:**
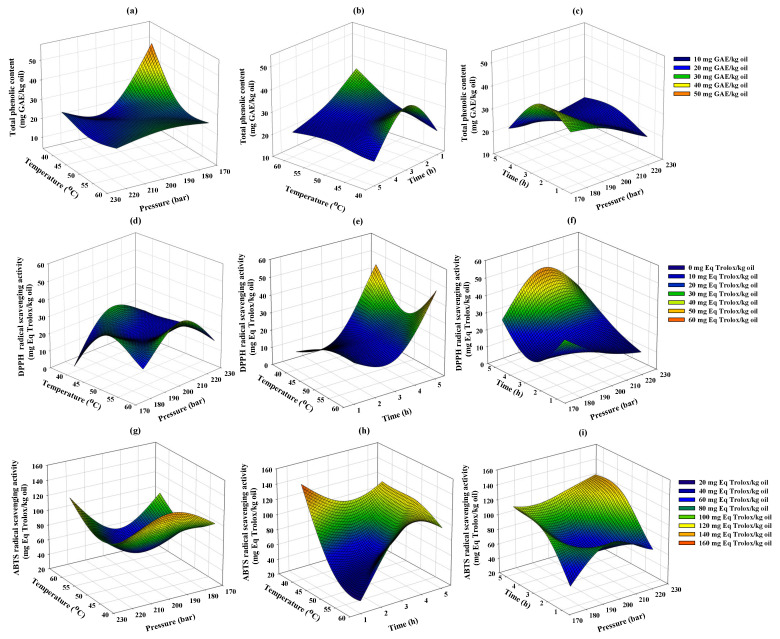
Response surface analysis showing the effects of extraction parameters including, temperature, pressure, and time on the (**a**–**c**) total phenolic content, (**d**–**f**) DPPH, and (**g**–**i**) ABTS radical scavenging activity of supercritical CO_2_-extracted cricket oil.

PV measures the amount of peroxide compounds, primarily hydroperoxides, which are the primary oxidation products formed during the early stages of lipid oxidation [37]. It is used as an indicator of oil freshness and to assess the oxidative stability of fats and oils. In this study, the PVs of SC-CO_2_-extracted cricket oils ranged from 20.06 to 70.34 mEq O_2_/kg oil, which was significantly higher than screw-pressed commercial cricket oil (8.22 mEq O_2_/kg oil). These results show that the oil extracted under SC-CO_2_ may undergo preliminary lipid oxidation under higher temperatures and pressures. Figure 2d–f illustrate the effect of extraction parameters on PV. PV increased as temperature increased from 40 to 50 °C, but declined as temperature increased to 60 °C. This is possibly due to higher extraction temperatures increasing the vapor pressure of phenolic compounds, leading to a reduction in PV [38,39]. PV was observed to increase as pressure increased but slightly decreased with longer extraction times, indicating that more natural antioxidants, such as polyphenols, were extracted over time, as shown in Figure 3.

#### 3.4.3. Iodine Value

IV reflects the degree of unsaturation in oil [40]. For cricket oil, statistical analysis showed no significant difference in IVs between SC-CO_2_-extracted oil (70.59 to 77.15 g I_2_/100 g oil) and screw-pressed commercial cricket oil (72.99 g I_2_/100 g oil). Figure 2g–i show that IVs slightly changed under different SC-CO_2_ extraction conditions. IV increased with increasing pressure and longer extraction time at constant temperature. This could be due to higher pressure improving SC-CO_2_ density and leading to selective extraction of unsaturated fatty acids [41]. At pressures lower than 200 bar, IV decreased as temperature increased, possibly due to a reduction in CO_2_ density, leading to lower solubility of unsaturated fatty acids and an increased proportion of saturated fatty acids [42].

#### 3.4.4. Saponification Value

SV the average molecular weight of fatty acids in oil. A higher SV suggests that the oil contains fatty acids with shorter carbon chains, while a lower SV suggests the presence of fatty acids with longer carbon chains [43]. SC-CO_2_-extracted cricket oils exhibited SV ranging from 178.07 to 196.76 mg KOH/g oil, which was comparable with screw-pressed commercial cricket oil (190.79 mg KOH/g oil). As presented in Figure 2j–l, SV was observed to increase as pressure increased at a constant temperature of 50 °C, possibly due to increased density of SC-CO_2_, which improved the solubility of short-chain fatty acids, resulting in higher SVs [38]. Moreover, SV slightly increased with increasing temperature at constant pressure, as higher temperatures increase the amount of free fatty acids in the oil, leading to high SVs [44].

#### 3.4.5. Specific Gravity of Extracted Cricket Oil Using Supercritical CO_2_

The physical properties, including specific gravity, viscosity, and color, are presented in Table 6, comparing oil extracted using SC-CO_2_ with screw-pressed commercial cricket oil. According to Table 6, the specific gravity of SC-CO_2_-extraced cricket oil was 0.91–0.92 and showed no significant difference from that of screw-pressed commercial cricket oil (*p* < 0.05). The specific gravity obtained from SC-CO_2_ extraction exhibited higher values compared to previous studies in which cricket oils were extracted using solvent extraction and screw pressing (0.87 and 0.88) [45]. This higher specific gravity may be attributed to the ability of supercritical CO_2_ to extract a greater proportion of high-molecular-weight compounds, such as waxes and phospholipids [46,47].

#### 3.4.6. Viscosity of Extracted Cricket Oil Using Supercritical CO_2_

The viscosity of extracted cricket oil varied from 77.36 to 132.35 cP, with most values higher than that of screw-pressed commercial cricket oil at 78.34 cP. The difference may be attributed to the ability of SC-CO_2_ to extract viscous compounds such as waxes, phospholipids, and long-chain triglycerides [46,47]. Moreover, fatty acid composition also plays an important role, as higher proportions of SFAs and MUFAs increase viscosity, while PUFAs reduce it due to their structural characteristics, leading to weaker intermolecular interactions [48,49]. In SC-CO_2_ extraction, extraction parameters including temperature, pressure, and time can influence the viscosity of the extracted oil. At a lower extraction temperature of 40 °C, oil viscosity increased at higher pressure due to the extraction of heavier components such as SFAs, long-chain triglycerides, and waxes. However, extraction time showed a different trend. During the initial stage of extraction, these heavier fractions were extracted first, resulting in higher viscosity. As the extraction time increased, lighter components such as PUFAs were increasingly extracted, leading to a decrease in oil viscosity. At a moderate temperature of 50 °C, oil viscosity slightly decreased with longer extraction time at lower pressure (175 bar). However, at higher pressure of 225 bar, oil viscosity was observed to increase with longer extraction time, suggesting that heavier components such as saturated fatty acids, long-chain triglycerides, and waxes were increasingly extracted over time. At a higher extraction temperature of 60 °C, viscosity slightly decreased at higher pressure, and at constant pressure, oil viscosity decreased over time, indicating increased extraction of PUFAs, which led to a reduction in oil viscosity. Overall, these results indicate that temperature influences the specific compounds extracted, while extraction time affects changes in the composition of the extracted oil throughout the process.

#### 3.4.7. Color Measurement of Extracted Cricket Oil Using Supercritical CO_2_

The color of SC-CO_2_-extracted cricket oil differed from that of screw-pressed commercial cricket oil (as shown in Table 6). SC-CO_2_-extracted oils generally exhibited brighter color (L*: 53.68 to 81.34 vs. 74.74), less redness (a*: 8.00 to 16.39 vs. 16.85), and more yellowness (b*: 88.75 to 124.96 vs. 112.80) compared to screw-pressed commercial cricket oil. The lighter yellow color can be attributed to the extraction mechanism, as SC-CO_2_ prefers to extract non-polar compounds under mild conditions [50], while limiting the extraction of polar compounds, phospholipids, and oxidation products. In contrast, screw-pressed extraction products may be contaminated with other co-extracted compounds and darker pigments [51]. The yellowness of cricket oil can be caused by the presence of lutein, canthaxanthin, β-cryptoxanthin, and β-carotene [52].

#### 3.4.8. Smoke Point of Cricket Oil

The smoke point is used as an indicator of thermal stability in edible oils and is used to determine its suitability for applications. When oil is heated above its smoke point, it can generate harmful substances, including short-chain fatty acids, trans fats, acrylamides, and polycyclic aromatic hydrocarbons [53]. The smoke points of cricket oil extracted with SC-CO_2_ were in a range of 144–150 °C obtained under conditions of 60 °C, 200 bar, for 5 h and 50 °C, 200 bar, for 3 h, which showed no significant difference in crude oil yield (*p* < 0.05). These values were higher than the screw-pressed commercial cricket oil (130 °C), as presented in Table 7. The lower smoke point of screw-pressed commercial cricket oil compared to SC-CO_2_-extracted oil may be due to its higher AV, which reflects a higher concentration of free fatty acids leading to the earlier formation of smoke [54]. According to their moderate smoke points (130–150 °C), cricket oils are not recommended for high-heat cooking methods such as deep frying or stir-frying, as they may easily produce smoke and undesirable compounds at higher temperatures [53]. Cricket oil has a fatty acid composition similar to that of edible rice bran oil, particularly in palmitic, stearic, oleic, and linoleic acids. The high smoke point of rice bran oil could be due to the low level of FFAs, high oxidative ability, presence of γ-oryzanol, and refined processing methods of commercial rice bran oil [55]. Although cricket oil has a lower smoke point than rice bran oil, it is also rich in oleic and linoleic acids, which are beneficial to human health.

### 3.5. Total Phenolic Content and Antioxidant Activity

TPC and antioxidant activities, including DPPH and ABTS radical scavenging activities, of extracted cricket oil using SC-CO_2_ were determined and compared with screw-pressed commercial cricket oil, as presented in Table 8. The variations in TPC and antioxidant properties under SC-CO_2_ extractions were further analyzed using RSM to investigate the influence of extraction parameters, including temperature, pressure, and time. The results are graphically presented using 3D surface plots in Figure 3.

#### 3.5.1. Total Phenolic Content

Phenolic compounds in oil play roles as antioxidants, cardiovascular disease preventatives, and anti-inflammatory agents [56]. In this study, the total phenolic content (TPC) of SC-CO_2_-extracted cricket oils ranged from 19.56 to 50.73 mg GAE/kg, while screw-pressed commercial cricket oil showed higher levels (91.41 mg GAE/kg). The higher TPC observed in screw-pressed oil may be attributed to the presence of fine solid particles and protein residues generated during the pressing process, which can be associated with phenolic compounds [57]. In contrast, supercritical CO_2_ extraction, due to its non-polar nature, predominantly extracts lipid-soluble phenolics [50].

As shown in Figure 3a–c, TPC decreased with increasing temperature and pressure at 40–50 °C but slightly increased at 50–60 °C, likely due to higher vapor pressure of phenolic compounds that may improve solubility of phenolic compounds in the SC-CO_2_ phase [39]. At constant extraction pressure, TPC decreased as extraction time increased. This reduction in TPC can be attributed to the selective nature of SC-CO_2_ for non-polar lipids [50], which limits the extraction of polar compounds such as phenolics. At constant extraction temperature, TPC initially decreased with increasing pressure during the early stage of extraction but gradually increased as extraction time increased. This suggests that longer extraction times may improve the extraction efficiency and enhance the solubility of phenolic compounds under these extraction conditions.

#### 3.5.2. DPPH Radical Scavenging Activity

The DPPH radical scavenging assay is generally used to quantify the antioxidant potential of lipophilic compounds by measuring their ability to neutralize free radicals [58]. DPPH radical scavenging activity of SC-CO_2_-extracted cricket oils ranged from 3.29 to 49.97 mg Eq Trolox/kg oil, while screw-pressed commercial cricket oil showed a relatively higher value of 42.35 mg Eq Trolox/kg oil, suggesting that screw pressing better preserves antioxidant compounds. As shown in Figure 3d–f, DPPH activity increased as pressure increased from 175 to 200 bar. This could be due to increased CO_2_ density and improved solubility of lipid-soluble antioxidants such as tocopherols [59]. At pressures above 200 bar, DPPH activity showed a slight reduction, possibly due to changes in solubility behavior under these conditions. As extraction time increased, DPPH slightly decreased at higher pressure, while TPC slightly increased. This behavior can be explained by the fact that not all phenolic compounds have the same antioxidant activity, and less active compounds may accumulate in the extract, leading to reduced DPPH activity.

#### 3.5.3. ABTS Radical Scavenging Activity

The ABTS radical scavenging assay measures antioxidant capacity in both hydrophilic and lipophilic compounds, enabling broader evaluation than DPPH [58]. In this study, the ABTS activity of SC-CO_2_-extracted cricket oils ranged from 36.82 to 145.90 mg Eq Trolox/kg oil, while screw-pressed commercial cricket oil exhibited higher activity (139.59 mg Eq Trolox/kg oil). This difference could be due to screw pressing retaining both hydrophilic and lipophilic antioxidants, whereas SC-CO_2_ prefers to extract lipid-soluble compounds, resulting in lower recovery of hydrophilic antioxidants such as phenolic compounds and leading to lower ABTS activity [60].

As shown in Figure 3g–i, ABTS activity slightly decreased as temperature increased from 40 to 50 °C but improved between 50 and 60 °C under higher pressure. This is possibly due to the lower solubility of antioxidants at lower extraction temperatures; however, as temperature and pressure increased, solvent power and mass transfer also increased, leading to greater antioxidant extraction. Although higher pressure consistently enhanced ABTS activity, longer extraction times slightly reduced it. This suggests that pressure plays a crucial role in enhancing the solubility and extraction efficiency of ABTS-related antioxidant compounds. The differing trends between DPPH and ABTS highlighted that different antioxidant compounds may dominate under varying extraction conditions.

Dried cricket, defatted cricket residue after oil extraction, supercritical CO_2_-extracted cricket oil, and screw-pressed commercial cricket oil were analyzed for phenolic compound types and contents (Table 9). Dried crickets and defatted residue contained gallic acid and myricetin as the main phenolics. Supercritical CO_2_-extracted cricket oil primarily contained gallic acid, whereas screw-pressed commercial cricket oil obtained via screw press showed gallic acid, myricetin, and trans-cinnamic acid as the major phenolics. The greater variety of phenolics in screw-pressed commercial cricket oil may be attributed to differences in extraction methods, as screw press extraction can retain more polar compounds, while supercritical CO_2_, being largely non-polar, might extract fewer polar compounds. This likely explains its higher total phenolic content and stronger DPPH and ABTS radical scavenging activities compared with supercritical CO_2_-extracted oil (Table 8).

### 3.6. Fatty Acids Composition

The fatty acid composition of house cricket oil obtained from SC-CO_2_ extraction and screw-pressed commercial cricket oil is presented in Table 10. Palmitic acid (C16:0), oleic acid (C18:1 n9c), linoleic acid (C18:2 n6c), and stearic acid (C18:0), which are major fatty acid constituents of neutral lipids, were predominant and consistent with previous reports [9,45,61]. In oil extraction using SC-CO_2_, it was observed that at extraction temperature of 40 °C, increasing pressure significantly enhanced the content of SFAs, resulting in a decrease in USFAs. However, at temperatures of 50–60 °C and pressures between 175 and 200 bar, SFAs exhibited an increasing trend but slightly decreased at 225 bar. These results suggest that temperature influences the types of compounds extracted, while extraction time affects how the composition of the oil changes during the process. The amounts of SFAs and USFAs in screw-pressed commercial cricket oil were within the ranges of oil obtained through oil extraction using SC-CO_2_. Cricket oil has the potential to be used as a fat source rich in oleic and linoleic acids. These fatty acids contribute to lowering LDL cholesterol and reducing cardiovascular risk [5,62]. Moreover, linoleic acid, as an essential omega-6 fatty acid, plays roles in skin health, inflammation control, and cellular function [63]. According to these results, SC-CO_2_-extracted cricket oil has significant potential as a valuable ingredient for further development in the food and cosmetics industries.

### 3.7. Vitamin E Content in Cricket Oil

Tocopherols (vitamin E) are essential antioxidants that protect polyunsaturated fatty acids, membrane components, and low-density lipoproteins from oxidation by free radicals [64]. In this study, SC-CO_2_-extracted cricket oils obtained at 60 °C and 200 bar for 5 h and at 50 °C and 200 bar for 3 h, which provided a high crude oil yield, were selected to determine vitamin E content. The oils contained 14.9 mg/100 g and 16.8 mg/100 g, respectively, which were lower than the previously reported value for house cricket oil (241 mg/100 g, including 75.3 mg/100 g of γ-tocopherol) [65]. However, when compared with animal fats, which are generally reported in terms of α-tocopherol content, SC-CO_2_-extracted cricket oil still exhibited higher relative content of vitamin E. For example, data from the USDA FoodData Central indicate that beef tallow contains approximately 2.7 mg/100 g of α-tocopherol, whereas lard contains about 0.6 mg/100 g of α-tocopherol [66,67]. These results indicate that SC-CO_2_-extracted cricket oil is a noteworthy source of vitamin E, which could be utilized to support nutritional applications and promote consumer health.

### 3.8. Cholesterol Content in Cricket Oil

Cholesterol is associated with cardiovascular disease. Cholesterol intake might increase the risk of vascular disease; however, the human body regulates its own cholesterol production, and individuals respond differently to cholesterol in their diet. Therefore, the relationship between dietary cholesterol and heart disease is still unclear [68]. In plants, only small amounts of cholesterol are found, and herbivorous insects are able to produce it by converting phytosterols [69]. Therefore, the cholesterol content of cricket oil was analyzed. In this study, SC-CO_2_-extracted cricket oil obtained at 60 °C and 200 bar for 5 h and at 50 °C and 200 bar for 3 h contained 1025 mg/100 g and 919.5 mg/100 g, respectively. These values were lower than the previously reported cholesterol content of house cricket oil (1510.81 mg/100 g) [70], which may be attributed to differences in feed composition. However, when compared with other fats, this level is substantially higher than those in tallow (109 mg/100 g) and lard (95 mg/100 g), while plant oils contain negligible cholesterol [66,67,71]. Nutritional recommendations also highlight the need for caution. The Thai Food and Drug Administration (FDA) recommends that Thai people limit their daily cholesterol intake to 300 mg or less [72]. However, in the Guidelines for Americans for the year 2020–2025, the specific limitation has been excluded, and it has been advised to consume dietary cholesterol as low as possible while maintaining a nutritionally adequate diet [73]. In conclusion, cricket oil is a rich source of beneficial unsaturated fatty acids, offering potential health advantages. However, its relatively high cholesterol content requires careful consumption, as excessive intake may elevate blood cholesterol levels and increase the risk of cardiovascular disease [68]. Moderation is therefore recommended to gain nutritional benefits while minimizing potential health risks.

### 3.9. Optimization and Verification of Cricket Oil Extraction Process Model

The optimal extraction condition for maximizing crude oil yield from crickets using SC-CO_2_ was 60 °C at 187.12 bar for 5 h, with an estimated yield of 16.36%. The model accuracy was validated by experiments conducted at a pressure point close to the predicted optimum, at 60 °C and 200 bar for 5 h. This condition was chosen to accommodate the limitations of the extraction equipment, as it was technically difficult to maintain the precise target pressure of 187.12 bar. Under these conditions, the experimental crude oil yield was 15.86%.

### 3.10. Study on Oxidative Stability of Cricket Oil

Cricket oil extracted under the validated optimal condition (60 °C, 200 bar for 5 h; Section 3.9) was selected to evaluate oxidative stability. Under this condition, the oil showed an AV of 4.25 mg KOH/g oil, a PV of 15.87 mEq O_2_/kg oil, an IV of 75.16 g I_2_/100 g oil, and an SV of 190.89 mg KOH/g oil. The TPC was 31.37 mg GAE/kg oil, while antioxidant activity was 37.21 mg Trolox equivalents/kg oil (DPPH) and 114.12 mg Trolox equivalents/kg oil (ABTS). Considering its low AV, moderate PV, notable antioxidant activity, and high unsaturation, this condition was selected for oxidative stability evaluation, as a low AV reflects reduced hydrolytic degradation and improved oxidative quality [74,75]. These combined properties are desirable for maintaining oil quality during storage. Therefore, the oil extracted at 60 °C, 200 bar, and 5 h was selected for further investigation of its oxidative stability.

**Figure 4:**
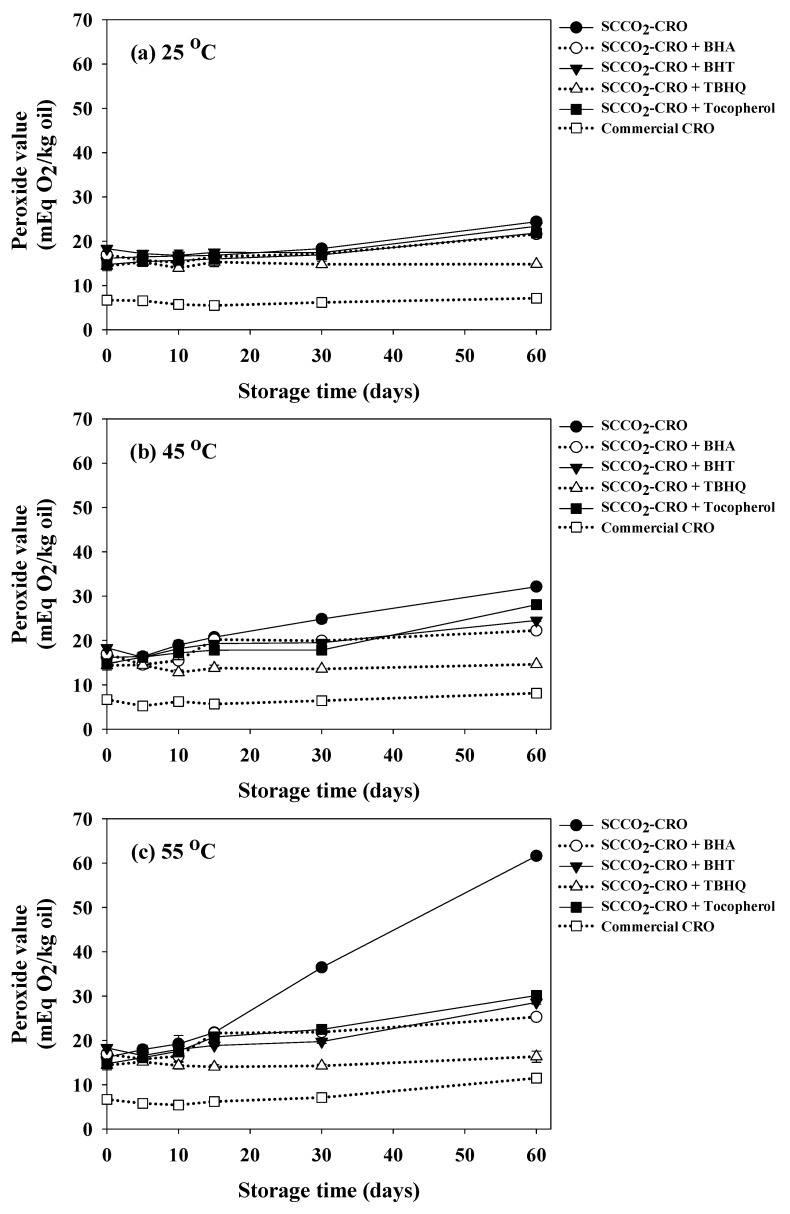
Changes in peroxide value of screw-pressed commercial cricket oil and supercritical CO_2_-extracted cricket oil treated with different food-grade antioxidants (BHA, BHT, TBHQ, and DL-α-tocopherol at 75 mg/kg) during storage at (**a**) 25 °C, (**b**) 45 °C, and (**c**) 55 °C for 60 days.

PV was monitored to study the oxidative stability of SC-CO_2_-extracted (with and without added antioxidants) compared with screw-pressed commercial cricket oils for 60 days of storage at 25, 45, and 55 °C as shown in Figure 4. Antioxidants, including BHA, BHT, TBHQ, and DL-α-tocopherol, were added in SC-CO_2_-extracted oil with the concentration of 75 mg/kg. The initial PV SC-CO_2_-extracted oil was 15.87 mEq O_2_/kg oil, which already exceeded the Codex limit (15 mEq O_2_/kg oil) [36]. PV of SC-CO_2_-extracted oil increased significantly with storage temperature and time, reaching 24.39, 32.13, and 61.62 mEq O_2_/kg oil at 25 °C, 45 °C, and 55 °C, respectively, after 60 days. In contrast, oil with added antioxidants showed the potential to retard lipid oxidation. Among the antioxidants, TBHQ was the most effective, maintaining PVs as low as 14.82 and 14.63 mEq O_2_/kg oil at 25 and 45 °C and delaying peroxide formation even at 55 °C (and 16.32 mEq O_2_/kg oil) after 60 days. This outstanding performance can be explained by its molecular structure, which results in strong radical scavenging capacity and high thermal stability [76,77]. BHA and BHT also improved stability, while DL-α-tocopherol provided moderate protection, especially at lower temperatures. In comparison, screw-pressed commercial cricket oil exhibited the lowest PVs (7.12, 8.11, and 11.47 mEq O_2_/kg oil) after 60 days, remaining within Codex limits across all storage conditions. The lower PVs may attributed to refining processes or natural antioxidants. Overall, TBHQ demonstrated the strongest antioxidant effect, followed by BHA, BHT, and DL-α-tocopherol, confirming that synthetic antioxidants can improve the oxidative stability of cricket oil.

## 4. Conclusions

This study demonstrated that supercritical CO_2_ (SC-CO_2_) extraction is an effective method for recovering cricket oil (*A. domesticus*). Extraction at 40–60 °C, 175–225 bar, and 1–5 h yielded 9.35–16.19% oil, with pressure and time positively influencing yield, while temperature had a slight negative effect. The SC-CO_2_-extracted oil exhibited acid values of 2.45–5.14 mg KOH/g, comparable iodine and saponification values to screw-pressed commercial cricket oil, and higher peroxide values, indicating a greater extent of lipid oxidation under certain extraction conditions. The major fatty acids were palmitic acid (27.36–28.84%), oleic acid (25.00–30.23%), stearic acid (6.81–8.53%), and linoleic acid (27.02–34.96%). The extracted oil showed higher viscosity than screw-pressed commercial cricket oil, similar specific gravity, and comparable color characteristics. Optimal extraction conditions were determined as 60 °C, 200 bar, and 5 h, achieving a maximum yield of 15.86%. Oxidative stability tests revealed that TBHQ was the most effective antioxidant under accelerated storage, followed by BHA, BHT, and DL-α-tocopherol, whereas SC-CO_2_ oil without antioxidants exhibited higher peroxide values. The SC-CO_2_-extracted cricket oil obtained in this study was rich in vitamin E but also contained a significant amount of cholesterol. This information is valuable for guiding its consumption, which should remain within recommended limits to minimize potential health risks. Overall, these findings highlight the potential of SC-CO_2_ extraction as an efficient and sustainable method for obtaining cricket oil for potential functional lipid applications.

## Figures and Tables

**Table 1:** Experimental treatment designed using the Box–Behnken design.

Treatment	Uncoded Independent Variables			Coded Independent Variables		
	Temperature (X_1_, °C)	Pressure (X_2_, bar)	Time (X_3_, h)	Temperature	Pressure	Time
1	40	175	3	−1	−1	0
2	60	175	3	1	−1	0
3	40	225	3	−1	1	0
4	60	225	3	1	1	0
5	40	200	1	−1	0	−1
6	60	200	1	1	0	−1
7	40	200	5	−1	0	1
8	60	200	5	1	0	1
9	50	175	1	0	−1	−1
10	50	225	1	0	1	−1
11	50	175	5	0	−1	1
12	50	225	5	0	1	1
13	50	200	3	0	0	0
14	50	200	3	0	0	0
15	50	200	3	0	0	0

**Table 2:** Physical and chemical properties of cricket oil samples obtained by Soxhlet extraction.

Physical and Chemical Properties	Cricket Oil Extracted by Soxhlet Extraction Method
% oil yield	15.75 ± 1.07
Acid value (mg KOH/g oil)	11.54 ± 0.15
Peroxide value (mEq O_2_/kg oil)	40.04 ± 2.57
Iodine value (g I_2_/100 g oil)	68.75 ± 2.09
Saponification value (mg KOH/g oil)	168.41 ± 2.63
Viscosity (cP)	64.18 ± 0.41
Density (g/cm^3^)	0.901 ± 0.001

**Table 3:** Percentage of crude oil yield, defatted cricket meal, and total mass balance from SC-CO_2_ extraction of cricket oil.

Extraction Conditions			Crude Oil Yield (%)	Defatted Cricket Meal (%)	Total Mass Balance (%)
Temperature (X_1_, °C)	Pressure (X_2_, bar)	Time (X_3_, h)			
40	175	3	13.58 ± 0.00 ^ef^	87.17 ± 0.95 ^abc^	100.75 ± 0.95 ^bcde^
60	175	3	14.24 ± 0.01 ^cde^	84.79 ± 0.52 ^ef^	99.02 ± 0.52 ^f^
40	225	3	15.21 ± 0.34 ^b^	86.24 ± 0.06 ^cd^	101.45 ± 0.39 ^ab^
60	225	3	15.34 ± 0.41 ^ab^	84.35 ± 0.91 ^fg^	99.70 ± 0.50 ^ef^
40	200	1	13.28 ± 1.19 ^f^	87.69 ± 0.06 ^a^	100.96 ± 1.13 ^bcd^
60	200	1	13.87 ± 0.67 ^def^	86.44 ± 0.69 ^bcd^	100.31 ± 0.03 ^cde^
40	200	5	15.14 ± 0.00 ^bc^	85.79 ± 0.00 ^de^	100.93 ± 0.00 ^bcd^
60	200	5	16.19 ± 0.28 ^a^	84.94 ± 0.04 ^ef^	101.13 ± 0.25 ^abc^
50	175	1	9.35 ± 0.00 ^g^	87.15 ± 0.00 ^abc^	96.50 ± 0.00 ^h^
50	225	1	14.66 ± 0.00 ^bcd^	87.39 ± 0.00 ^ab^	102.05 ± 0.00 ^a^
50	175	5	15.54 ± 0.19 ^ab^	83.66 ± 0.17 ^g^	99.20 ± 0.03 ^f^
50	225	5	13.83 ± 0.00 ^def^	84.01 ± 0.00 ^fg^	97.84 ± 0.00 ^g^
50	200	3	15.42 ± 0.23 ^ab^	85.06 ± 0.66 ^ef^	100.48 ± 0.43 ^bcde^
50	200	3	15.01 ± 0.10 ^bc^	84.91 ± 0.36 ^ef^	99.92 ± 0.26 ^def^
50	200	3	15.12 ± 0.35 ^bc^	84.89 ± 0.04 ^ef^	100.01 ± 0.31 ^def^

Note: Means in the same column with different superscript letters are statistically significantly different at the 95% confidence level (*p* < 0.05).

**Table 4:** Analysis of variance (ANOVA) for the polynomial model describing the crude oil yield obtained from SC-CO_2_.

Source	DF	Seq SS	Adj SS	Adj MS	F	*p*
Model	7	72.243	72.243	10.321	63.440	0.000
Linear	3	34.296	34.296	11.432	70.280	0.000
Temperature	1	1.479	1.479	1.479	9.090	0.006
Pressure	1	10.042	10.042	10.042	61.730	0.000
Time	1	22.775	22.775	22.775	140.000	0.000
Square	3	13.299	13.299	4.433	27.250	0.000
Temperature × Temperature	1	1.673	0.863	0.863	5.310	0.031
Pressure × Pressure	1	5.541	6.435	6.435	39.560	0.000
Time × Time	1	6.084	6.084	6.084	37.400	0.000
Interaction	1	24.649	24.649	24.649	151.520	0.000
Pressure × Time	1	24.649	24.649	24.649	151.520	0.000
Residual Error	22	3.579	3.579	0.163		
Lack-of-Fit	5	0.959	0.959	0.192	1.240	0.332
Pure Error	17	2.620	2.620	0.154		
Total	29	75.822				

R^2^_adj_. = 93.78%.

**Table 5:** Physicochemical properties of cricket oil extracted using supercritical CO_2_ compared with screw-pressed commercial cricket oil.

Temperature (°C)	Pressure (bar)	Time (h)	Acid Value (mg KOH/g Oil)	Peroxide Value (mEq O_2_/kg Oil)	Iodine Value ^ns^ (g I_2_/100 g Oil)	Saponification Value (mg KOH/g Oil)
40	175	3	4.76 ± 0.96 ^ab^	20.06 ± 1.83 ^j^	74.80 ± 2.32	178.07 ± 8.33 ^c^
60	175	3	3.71 ± 0.18 ^cde^	25.42 ± 0.03 ^i^	71.06 ± 5.53	196.76 ± 8.80 ^a^
40	225	3	2.97 ± 0.39 ^defg^	38.87 ± 1.11 ^g^	76.20 ± 1.02	189.10 ± 8.60 ^abc^
60	225	3	3.39 ± 0.17 ^cdef^	67.82 ± 0.09 ^a^	75.33 ± 1.31	193.90 ± 6.73 ^ab^
40	200	1	3.98 ± 0.21 ^bc^	32.88 ± 1.10 ^h^	76.19 ± 1.43	191.59 ± 4.48 ^abc^
60	200	1	5.14 ± 0.80 ^a^	23.36 ± 1.57 ^i^	72.68 ± 4.33	191.05 ± 7.24 ^abc^
40	200	5	3.18 ± 0.06 ^cdefg^	50.94 ± 1.78 ^d^	70.59 ± 5.02	182.31 ± 0.12 ^bc^
60	200	5	2.91 ± 0.32 ^efg^	47.29 ± 0.39 ^e^	75.62 ± 0.62	186.46 ± 1.45 ^abc^
50	175	1	4.79 ± 0.03 ^ab^	44.03 ± 0.80 ^f^	70.94 ± 4.41	181.98 ± 8.21 ^bc^
50	225	1	2.45 ± 0.05 ^g^	70.34 ± 0.82 ^a^	75.01 ± 3.51	188.97 ± 0.21 ^abc^
50	175	5	3.25 ± 0.08 ^cdefg^	32.35 ± 0.58 ^h^	75.46 ± 0.26	190.39 ± 3.85 ^abc^
50	225	5	2.76 ± 0.03 ^fg^	38.76 ± 1.60 ^g^	77.15 ± 0.40	183.74 ± 3.91 ^abc^
50	200	3	3.33 ± 0.23 ^cdef^	56.61 ± 1.29 ^c^	75.40 ± 1.57	192.93 ± 3.52 ^ab^
50	200	3	3.82 ± 0.29 ^cd^	57.61 ± 0.72 ^c^	74.96 ± 1.48	191.10 ± 3.04 ^abc^
50	200	3	3.54 ± 0.27 ^cdef^	63.94 ± 4.02 ^b^	73.80 ± 1.24	184.57 ± 3.59 ^abc^
Screw-pressed commercial cricket oil			5.17 ± 0.03 ^a^	8.22 ± 0.41 ^k^	72.99 ± 5.99	190.79 ± 5.15 ^abc^

**Note:** Means in the same column with different superscript letters are statistically significantly different at the 95% confidence level (*p* < 0.05). ns stands for not statistically significant.

**Table 6:** Viscosity, specific gravity, and color parameters of cricket oil extracted using SC-CO_2_.

Temperature (°C)	Pressure (bar)	Time (h)	Specific Gravity	Viscosity (cP)		Color Parameters	
					L*	a*	b*
40	175	3	0.91 ± 0.00 ^b^	109.45 ± 0.35 ^bc^	56.99 ± 0.28 ^ij^	11.49 ± 0.03 ^i^	90.43 ± 0.38 ^kl^
60	175	3	0.91 ± 0.00 ^b^	101.80 ± 0.57 ^de^	65.71 ± 1.06 ^f^	11.22 ± 0.13 ^j^	104.93 ± 1.39 ^g^
40	225	3	0.91 ± 0.00 ^b^	132.35 ± 5.30 ^a^	70.51 ± 2.14 ^e^	13.75 ± 0.37 ^e^	113.39 ± 3.09 ^e^
60	225	3	0.92 ± 0.00 ^ab^	101.00 ± 0.57 ^e^	61.58 ± 0.52 ^g^	14.57 ± 0.08 ^d^	100.77 ± 0.66 ^h^
40	200	1	0.91 ± 0.00 ^b^	81.81 ± 1.38 ^hi^	72.85 ± 1.29 ^d^	12.25 ± 0.21 ^g^	116.25 ± 1.76 ^d^
60	200	1	0.91 ± 0.00 ^b^	91.81 ± 0.59 ^g^	57.69 ± 1.09 ^i^	10.49 ± 0.02 ^k^	93.35 ± 1.57 ^j^
40	200	5	0.91 ± 0.00 ^b^	77.36 ± 1.37 ^j^	78.32 ± 0.11 ^b^	9.55 ± 0.05 ^l^	122.48 ± 0.23 ^b^
60	200	5	0.91 ± 0.00 ^b^	83.89 ± 1.17 ^h^	81.34 ± 0.76 ^a^	8.00 ± 0.19 ^m^	124.96 ± 1.05 ^a^
50	175	1	0.91 ± 0.00 ^b^	97.07 ± 0.62 ^f^	66.28 ± 0.22 ^f^	11.69 ± 0.06 ^hi^	106.17 ± 0.37 ^fg^
50	225	1	0.91 ± 0.00 ^b^	92.08 ± 1.77 ^g^	55.86 ± 0.47 ^j^	13.29 ± 0.02 ^f^	91.71 ± 0.70 ^jk^
50	175	5	0.91 ± 0.00 ^b^	81.81 ± 1.38 ^hi^	75.85 ± 1.54 ^c^	11.75 ± 0.23 ^h^	120.15 ± 2.13 ^c^
50	225	5	0.92 ± 0.00 ^a^	105.00 ± 0.42 ^d^	66.14 ± 0.43 ^f^	16.39 ± 0.08 ^b^	107.86 ± 0.55 ^f^
50	200	3	0.91 ± 0.00 ^b^	109.85 ± 0.21 ^bc^	59.93 ± 0.75 ^h^	16.17 ± 0.06 ^bc^	98.36 ± 1.16 ^i^
50	200	3	0.91 ± 0.00 ^b^	108.75 ± 0.64 ^c^	53.68 ± 0.09 ^k^	14.78 ± 0.01 ^d^	88.75 ± 0.13 ^l^
50	200	3	0.92 ± 0.00 ^ab^	112.50 ± 0.85 ^b^	60.03 ± 0.33 ^h^	15.97 ± 0.01 ^c^	98.76 ± 0.38 ^hi^
Screw-pressed commercial cricket oil			0.92 ± 0.00 ^a^	78.34 ± 0.39 ^ij^	74.74 ± 0.10 ^c^	16.85 ± 0.06 ^a^	112.80 ± 0.13 ^e^

**Note:** Means in the same column with different superscript letters are statistically significantly different at the 95% confidence level (*p* < 0.05).

**Table 7:** Smoke point characteristics of cricket oil.

Temperature (°C)	Pressure (bar)	Time (h)	Smoke Point (°C)
60	200	5	144.00 ± 4.24 ^c^
50	200	3	150.00 ± 4.24 ^b^
Screw-pressed commercial cricket oil			130.00 ± 2.83 ^d^
Rice bran oil			216.00 ± 1.42 ^a^

**Note:** Means in the same column with different superscript letters are statistically significantly different at the 95% confidence level (*p* < 0.05).

**Table 8:** Total phenolic content, DPPH, and ABTS radical scavenging activity of supercritical CO_2_-extracted cricket oil.

Temperature (°C)	Pressure (bar)	Time (h)	Total Phenolic Content (mg GAE/kg Oil)	DPPH (mg Eq Trolox/kg Oil)	ABTS (mg Eq Trolox/kg Oil)
40	175	3	50.73 ± 0.47 ^b^	3.29 ± 0.11 ^k^	106.17 ± 4.14 ^cde^
60	175	3	27.17 ± 1.14 ^ef^	18.68 ± 0.06 ^gh^	103.88 ± 5.40 ^de^
40	225	3	25.71 ± 1.00 ^fg^	19.10 ± 0.05 ^gh^	111.67 ± 1.20 ^cd^
60	225	3	25.67 ± 1.55 ^fg^	17.52 ± 0.14 ^i^	124.57 ± 2.96 ^b^
40	200	1	20.83 ± 0.84 ^ij^	10.32 ± 1.73 ^j^	145.90 ± 7.77 ^a^
60	200	1	37.17 ± 0.68 ^c^	23.32 ± 0.38 ^f^	36.82 ± 1.97 ^i^
40	200	5	20.81 ± 1.45 ^ij^	47.58 ± 0.91 ^b^	120.84 ± 1.95 ^b^
60	200	5	21.18 ± 1.02 ^ij^	49.97 ± 0.56 ^a^	100.56 ± 1.98 ^e^
50	175	1	33.44 ± 1.63 ^d^	32.35 ± 1.82 ^d^	48.97 ± 1.70 ^h^
50	225	1	19.56 ± 0.58 ^k^	9.39 ± 1.52 ^j^	65.07 ± 0.23 ^f^
50	175	5	21.76 ± 0.29 ^i^	26.39 ± 0.08 ^e^	112.93 ± 2.64 ^c^
50	225	5	24.19 ± 0.67 ^gh^	31.66 ± 1.29 ^d^	125.85 ± 1.87 ^b^
50	200	3	24.59 ± 0.51 ^gh^	18.32 ± 0.65 ^i^	59.48 ± 1.73 ^fg^
50	200	3	22.75 ± 0.50 ^hi^	18.26 ± 0.54 ^i^	56.04 ± 1.27 ^gh^
50	200	3	28.04 ± 0.34 ^e^	20.43 ± 0.86 ^g^	63.13 ± 1.73 ^fg^
Screw-pressed commercial cricket oil			91.41 ± 0.80 ^a^	42.35 ± 0.59 ^c^	139.59 ± 6.86 ^a^

**Note:** Means in the same column with different superscript letters are statistically significantly different at the 95% confidence level (*p* < 0.05).

**Table 9:** Phenolic compounds in dried cricket, defatted cricket, supercritical CO_2_-extracted cricket oil, and screw-pressed commercial cricket oil.

Samples	Phenolic Compounds	Content
Dried cricket	Gallic acid (µg/g)	1.30 ± 0.07
	Myricetin (µg/g)	2.64 ± 0.36
Defatted cricket from supercritical CO_2_ extraction (50 °C, 200 bar, 3 h)	Gallic acid (µg/g)	0.56 ± 0.00
	Myricetin (µg/g)	0.93 ± 0.01
Defatted cricket from supercritical CO_2_ extraction (60 °C, 175 bar, 5 h)	Gallic acid (µg/g)	0.35 ± 0.09
	Myricetin (µg/g)	1.72 ± 0.08
Cricket oil from supercritical CO_2_ extraction (50 °C, 200 bar, 3 h)	Gallic acid (µg/mL)	2.55 ± 0.02
Cricket oil from supercritical CO_2_ extraction (60 °C, 175 bar, 5 h)	Gallic acid (µg/mL)	2.50 ± 0.05
Cricket oil from supercritical CO_2_ extraction (60 °C, 200 bar, 5 h)	Gallic acid (µg/mL)	4.69 ± 0.20
Screw-pressed commercial cricket oil	Gallic acid (µg/mL)	3.51 ± 0.12
	Myricetin (µg/mL)	28.62 ± 4.12
	Trans-cinnamic acid (µg/mL)	1.04 ± 0.24

**Table 10:** Fatty acid composition of cricket oil.

Fatty Acid Composition (%)	SC-CO_2_ Extraction Condition									Screw-Pressed Commercial Cricket Oil
	40 °C 175 bar 3 h	40 °C 200 bar 5 h	40 °C 225 bar 3 h	50 °C 175 bar 5 h	50 °C 200 bar 3 h	50 °C 225 bar 5 h	60 °C 175 bar 3 h	60 °C 200 bar 5 h	60 °C 225 bar 3 h	
Lauric acid (C12:0)	0.11 ± 0.01 ^ab^	0.12 ± 0.00 ^a^	0.10 ± 0.01 ^abc^	0.11 ± 0.01 ^ab^	0.09 ± 0.01 ^cd^	0.11 ± 0.00 ^ab^	0.10 ± 0.00 ^bcd^	nd	0.09 ± 0.00 ^d^	nd
Myristic acid (C14:0)	0.57 ± 0.01 ^cd^	0.53 ± 0.00 ^e^	0.61 ± 0.01 ^ab^	0.56 ± 0.00 ^de^	0.54 ± 0.02 ^e^	0.54 ± 0.00 ^e^	0.59 ± 0.00 ^bcd^	0.54 ± 0.01 ^e^	0.62 ± 0.00 ^a^	0.59 ± 0.03 ^abc^
Pentadecylic acid (C15:0)	0.09 ± 0.01 ^bc^	0.10 ± 0.00 ^bc^	0.13± 0.03 ^a^	0.11 ± 0.01 ^b^	0.09 ± 0.00 ^bc^	0.10 ± 0.00 ^bc^	0.09 ± 0.00 ^bc^	0.09 ± 0.01 ^bc^	0.08 ± 0.00 ^c^	0.06 ± 0.00 ^d^
Palmitic acid (C16:0)	27.84 ± 0.01 ^cd^	27.81 ± 0.02 ^cd^	27.87 ± 0.05 ^c^	27.91 ± 0.01 ^c^	28.84 ± 0.01 ^a^	27.36 ± 0.00 ^d^	28.27 ± 0.03 ^bc^	28.50 ± 0.11 ^ab^	28.31 ± 0.02 ^bc^	28.30 ± 0.05 ^bc^
Palmitoleic acid (C16:1)	0.72 ± 0.05 ^de^	0.67 ± 0.00 ^ef^	0.99 ± 0.03 ^a^	0.63 ± 0.01 ^f^	0.67 ± 0.00 ^ef^	0.66 ± 0.00 ^f^	0.75 ± 0.00 ^cd^	0.74 ± 0.00 ^cd^	0.78 ± 0.00 ^bc^	0.81 ± 0.03 ^b^
Margaric acid (C17:0)	0.26 ± 0.03 ^abc^	0.29 ± 0.00 ^abc^	0.31 ± 0.00 ^a^	0.29 ± 0.00 ^ab^	0.28 ± 0.00 ^abc^	0.27 ± 0.00 ^bcd^	0.24 ± 0.00 ^de^	0.26 ± 0.01 ^cd^	0.22 ± 0.00 ^e^	0.16 ± 0.03 ^f^
Stearic acid (C18:0)	6.95 ± 0.42 ^de^	6.81 ± 0.02 ^e^	7.09 ± 0.04 ^de^	7.50 ± 0.00 ^c^	8.10 ± 0.01 ^b^	6.99 ± 0.00 ^de^	7.26 ± 0.02 ^cd^	8.17 ± 0.0 ^b^	8.11 ± 0.03 ^b^	8.53 ± 0.01 ^a^
Elaidic acid (C18:1 n9t)	nd	nd	2.00 ± 0.07 ^a^	0.92 ± 0.00 ^c^	0.83 ± 0.01 ^d^	nd	nd	1.13 ± 0.01 ^b^	nd	0.72 ± 0.07 ^e^
Oleic acid (C18:1 n9c)	28.22 ± 0.78 ^c^	27.16 ± 0.06 ^ef^	25.00 ± 0.01 ^g^	27.31 ± 0.00 ^def^	27.68 ± 0.03 ^cde^	27.83 ± 0.03 ^cd^	28.84 ± 0.08 ^b^	26.82 ± 0.03 ^f^	30.23 ± 0.03 ^a^	28.79 ± 0.03 ^b^
Linoelaidic acid (C18:2 n6t)	nd	nd	0.10 ± 0.01 ^a^	0.09 ± 0.00 ^b^	0.08 ± 0.00 ^c^	nd	nd	nd	nd	nd
Linoleic acid (C18:2 n6c)	33.68 ± 1.88 ^a^	34.96 ± 0.00 ^a^	27.02 ± 0.01 ^e^	32.04 ± 0.00 ^b^	30.48 ± 0.02 ^c^	34.55 ± 0.01 ^a^	32.31 ± 0.06 ^b^	28.38 ± 0.04 ^d^	30.07 ± 0.01 ^c^	30.05 ± 0.06 ^c^
Arachidic acid (C20:0)	0.26 ± 0.03 ^c^	0.30 ± 0.00 ^b^	0.31 ± 0.02 ^b^	0.34 ± 0.00 ^a^	0.34 ± 0.00 ^a^	0.30 ± 0.01 ^b^	0.24 ± 0.00 ^c^	0.31 ± 0.01 ^b^	0.24 ± 0.00 ^c^	0.24 ± 0.00 ^c^
γ-Linolenic acid (C18:3 n6)	nd	nd	0.50 ± 0.00 ^a^	0.07 ± 0.01 ^c^	0.06 ± 0.00 ^d^	nd	nd	0.24 ± 0.00 ^b^	nd	0.06 ± 0.00 ^d^
Gondoic acid (C20:1)	nd	nd	nd	0.05 ± 0.01 ^b^	0.05 ± 0.00 ^b^	nd	nd	0.09 ± 0.02 ^a^	nd	0.03 ± 0.00 ^c^
α-Linolenic acid (C18:3 n3)	0.63 ± 0.05 ^bc^	0.58 ± 0.01 ^d^	0.41 ± 0.01 ^f^	0.52 ± 0.02 ^e^	0.52 ± 0.00 ^e^	0.60 ± 0.01 ^cd^	0.67 ± 0.01 ^ab^	0.64 ± 0.00 ^bc^	0.69 ± 0.00 ^a^	0.49 ± 0.02 ^e^
Heneicosylic acid (C21:0)	0.19 ± 0.01 ^a^	0.14 ± 0.00 ^d^	nd	nd	nd	0.15 ± 0.00 ^c^	0.20 ± 0.00 ^a^	nd	0.18 ± 0.00 ^b^	nd
Eicosadienoic acid (C20:2)	0.24 ± 0.02 ^fg^	0.25 ± 0.00 ^f^	2.25 ± 0.02 ^a^	0.52 ± 0.01 ^c^	0.49 ± 0.01 ^d^	0.25 ± 0.00 ^f^	0.26 ± 0.00 ^f^	1.31 ± 0.01 ^b^	0.22 ± 0.00 ^g^	0.43 ± 0.01 ^e^
Behenic acid (C22:0)	0.04 ± 0.01 ^d^	0.05 ± 0.00 ^c^	nd	0.11 ± 0.00 ^a^	0.10 ± 0.00 ^b^	0.06 ± 0.00 ^c^	0.04 ± 0.00 ^d^	0.10 ± 0.01 ^b^	0.03 ± 0.00 ^d^	0.04 ± 0.00 ^d^
Dihomo-γ-linolenic acid (C20:3 n6)	nd	nd	0.12 ± 0.02 ^a^	0.05 ± 0.00 ^b^	0.04 ± 0.00 ^b^	nd	nd	0.06 ± 0.00 ^b^	nd	0.02 ± 0.00 ^c^
Tricosylic acid (C23:0)	nd	nd	2.15 ± 0.00 ^a^	0.36 ± 0.00 ^c^	0.27 ± 0.00 ^d^	nd	nd	1.08 ± 0.03 ^b^	nd	0.26 ± 0.01 ^d^
Arachidonic acid (C20:4 n6)	0.10 ± 0.00 ^c^	0.11 ± 0.00 ^c^	2.15 ± 0.00 ^a^	0.08 ± 0.00 ^d^	0.08 ± 0.00 ^d^	0.10 ± 0.00 ^c^	0.10 ± 0.00 ^c^	0.15 ± 0.00 ^b^	0.08 ± 0.00 ^d^	0.04 ± 0.02 ^e^
Docosadienoic acid (C22:2)	0.02 ± 0.00 ^d^	0.03 ± 0.00 ^d^	1.64 ± 0.05 ^a^	0.23 ± 0.01 ^c^	0.20 ± 0.00 ^c^	0.02 ± 0.00 ^d^	0.02 ± 0.00 ^d^	0.81 ± 0.12 ^b^	0.02 ± 0.00 ^d^	0.20 ± 0.04 ^c^
Lignoceric acid (C24:0)	0.04 ± 0.01 ^efg^	0.05 ± 0.00 ^e^	0.43 ± 0.02 ^a^	0.11 ± 0.00 ^c^	0.10 ± 0.01 ^c^	0.05 ± 0.00 ^ef^	0.03 ± 0.00 ^fg^	0.29 ± 0.00 ^b^	0.02 ± 0.00 ^g^	0.08 ± 0.00 ^d^
Eicosapentaenoic acid (C20:5 n3)	0.03 ± 0.02 ^b^	0.05 ± 0.00 ^a^	nd	nd	nd	0.05 ± 0.00 ^a^	0.01 ± 0.00 ^bc^	nd	0.02 ± 0.00 ^b^	nd
Docosahexaenoic acid (C22:6 n3)	nd	nd	0.72 ± 0.01 ^a^	0.10 ± 0.00 ^c^	0.09 ± 0.01 ^c^	nd	nd	0.32 ± 0.0 ^b^	nd	0.09 ± 0.01 ^c^
Saturated fatty acids (SFAs)	36.37 ± 1.18 ^f^	36.20 ± 0.05 ^f^	39.00 ± 0.19 ^a^	37.40 ± 0.05 ^de^	38.74 ± 0.06 ^ab^	35.94 ± 0.02 ^f^	37.05 ± 0.07 ^e^	39.33 ± 0.20 ^a^	37.90 ± 0.06 ^cd^	38.26 ± 0.15 ^bc^
Monounsaturated fatty acids (MUFAs)	28.94 ± 0.83 ^de^	27.82 ± 0.06 ^g^	27.99 ± 0.11 ^fg^	28.91 ± 0.03 ^de^	29.23 ± 0.04 ^cd^	28.49 ± 0.03 ^ef^	29.58 ± 0.09 ^c^	28.77 ± 0.05 ^de^	31.01 ± 0.04 ^a^	30.36 ± 0.14 ^b^
Polyunsaturated fatty acids (PUFAs)	34.69 ± 1.97 ^bc^	35.98 ± 0.02 ^a^	33.01 ± 0.14 ^de^	33.69 ± 0.05 ^cd^	32.04 ± 0.04 ^ef^	35.57 ± 0.03 ^ab^	33.37 ± 0.07 ^d^	31.90 ± 0.18 ^ef^	31.09 ± 0.02 ^g^	31.38 ± 0.15 ^g^

**Note:** Means in the same column with different superscript letters are statistically significantly different at the 95% confidence level (*p* < 0.05); nd stands for not detected.

## Data Availability

The data presented in this study are available on request from the corresponding author. The raw data supporting the conclusions of this article will be made available by the authors on request.
